# Compact femtosecond fiber laser tunable from 800 to 850 nm with pulse energy exceeding 5 nJ

**DOI:** 10.1038/s41598-025-04063-8

**Published:** 2025-05-29

**Authors:** Dmitrii Stoliarov, Aleksandr Koviarov, Diana Galiakhmetova, Edik Rafailov

**Affiliations:** Aston Institute of Photonic Technologies, Aston University, B4 7ET Birmingham, UK

**Keywords:** High-harmonic generation, Fibre lasers, Mode-locked lasers, Ultrafast lasers

## Abstract

This paper introduces a compact, tunable femtosecond laser based on an Erbium-doped fiber, utilizing the Self-Soliton Frequency Shifted technique and PPLN crystal as a Second Harmonic Generation module. Achieving an unparalleled frequency conversion efficiency up to 55% for the 800 - 850 nm wavelength range, this compact laser emits sub-100 fs pulses. The laser operates simultaneously within the first and third biological windows, delivering pulse energies of 10.4 nJ and 5.1 nJ, respectively. This performance, previously unattained in similar systems, is achieved while maintaining Second Harmonic Generation power stability below 2% RMS. The presented compact laser, developed for bladder cancer detection through multiphoton microscopy, will significantly improve the system’s compactness, precision, and cancer detection efficiency.

## Introduction

In 2020, 10 million cancer deaths occurred, partly due to delayed diagnosis, with bladder cancer (BC) being notably prevalent, highlighting the need for improved detection methods^1^. Traditional techniques like White Light Imaging and narrow-band imaging have limitations, such as inadequate contrast and high reliance on physician interpretation, which can lead to false negative/positive results^2,3^. These issues are especially critical in detecting early-stage cancers.

Recent advances have been made in multiphoton imaging, a more promising method that uses high-power laser pulses and non-linear optics effects such as: second harmonic generation (SHG), third harmonic generation (THG), two-photon excited fluorescence (TPEF), three-photon excited fluorescence (3PEF), etc. By utilizing near-infrared (NIR) radiation in the spectral ranges of “biological windows” instead of visible or ultraviolet radiation, multiphoton imaging allows providing deep penetration through scattering tissues, diffraction-limited resolution with optical sectioning, and fast imaging speeds^4^.

Biological windows are so named because they allow for higher penetration depths of light into biological tissue within specific spectral regions ^5^. The first biological window, spanning 700 - 950 nm, has been extensively studied over recent years, primarily due to the wide use of Ti:Sapphire (Ti:Sa) lasers. These lasers offer wavelength tunability from 700 to 1080 nm with ultrashort pulse durations, making them ideal for applications within the first biological window. Additionally, the use of an optical parametric oscillator (OPO) extends their coverage to other biological windows, further expanding their potential applications^6^. In a study by Kurilchik S *et al.*, a multiphoton imaging system was proposed for breast cancer diagnostics^7^. A central component of this system was a tunable high-peak-power femtosecond laser operating at approximately 830 nm. This wavelength enables TPEF imaging of key metabolic indicators such as NADH and FAD, which are often sequentially excited at 750 nm and 880 nm, respectively^8^. Other intrinsic fluorophores relevant to cancer detection - including porphyrins, collagen, elastin, keratin, and lipofuscin - also exhibit strong two-photon absorption within this range, supporting the importance of ultrafast sources operating near 800 nm for functional and label-free biomedical imaging^9^.

In contrast, the third biological window (1600 - 1870 nm) remains relatively underexplored for multiphoton imaging but holds significant promise for deep tissue imaging, offering advantages over the first and second biological windows. For example, studies using 1700 nm excitation have demonstrated successful deep-brain imaging with a signal-to-noise ratio (SNR) of  5 dB and a penetration depth of 1.1 mm^10^. Similar results have been reported in the urinary system, where 1700 nm light achieved a penetration depth of $800 \upmu \hbox {m}$ in normal prostate tissue^11^. The enhanced penetration capability is attributed to reduced scattering at longer excitation wavelengths, which requires less power to reach the focus and significantly increases the depth of tissue penetration^10^. Furthermore, the nonlinear features of multiphoton microscopy help minimize out-of-focus excitation and sample photobleaching sample during imaging process^12^. Given the reduced scattering and increased penetration depth, the use of longer wavelengths for excitation and emission is highly advantageous for imaging systems^7^. Therefore, the first and third biological windows are particularly attractive regions for biomedical imaging. However, there remains a notable lack of commercial femtosecond lasers capable of operating within these spectral regions. Developing a laser source that generates femtosecond pulses within both biological windows could facilitate deeper tissue imaging, achieving penetration depths in the millimeter range for in vivo detection of bladder and gastrointestinal cancers. By delivering wavelengths through a single channel, such a system would enable higher-resolution, faster imaging with more accurate diagnostics. This advanced imaging capability is expected to significantly improve clinicians’ ability to diagnose and monitor treatment outcomes more efficiently^7,13^.

The potential for implementing Ti:Sa lasers to BC diagnostics medical systems is relatively low due to the laser complexity, high cost, large footprint, and susceptibility to vibrations and environmental perturbations^14^. Recent advancements, including SESAM-mode-locked, diode-pumped Ti:sapphire lasers, have significantly improved the compactness and pump efficiency of Ti:Sa systems^15^. However, SESAMs typically offer limited spectral tunability, have finite operational lifetimes under high-intensity exposure, and require precise alignment of the free-space cavity along with careful environmental stabilization. The alternatives to traditional laser systems include fiber lasers, which leverage nonlinear effects such as self-phase modulation (SPM) or Raman self-pumping, which culminate in self-soliton frequency shifting (SSFS), followed by frequency doubling^16–18^. While potentially more affordable and reliable, these systems are not yet commercially available and exhibit several limitations, including low conversion efficiency and unstable output power. By overcoming these challenges Wei L. *et al.* demonstrated an Yb-fiber laser with an SPM stage capable of delivering femtosecond pulses with tunable wavelengths from 920 nm to 1210 nm, achieving pulse energies of up to 10 nJ^19^. A complementary approach was presented by Resan B. et al., who employed a bulk Yb tungstate seed laser in combination with spectral broadening in a microstructured fiber and external pulse compression to generate 30 fs pulses tunable around $1\,\upmu \hbox {m}$^20^. Another approach was introduced by Dai R. *et al.*, who developed a tunable laser with a spectral range from 932 nm to 962 nm based on a Thulium fiber laser paired with a periodically poled lithium niobate (PPLN) frequency-doubling crystal. They demonstrated SHG pulses of 95 fs duration and energies of 42 nJ^21^, achieving a conversion efficiency for the SHG process of 52%. Additionally, they applied this laser in a two-photon microscope, obtaining commercial-grade results. Periodically and aperiodically poled potassium titanyl phosphate (KTP) are extensively utilized in both bulk and waveguide forms for SHG, notably for high-efficiency infrared-to-blue frequency conversion^22,23^. However, it is PPLN crystals that have demonstrated exceptional performance, with single-pass internal conversion efficiencies reaching 70% for generating 775 nm in 50 mm long ridge waveguides^24^. This highlights the capability of PPLN in the domain of effective frequency conversion.

Charan K. *et al.* utilized Er-fiber lasers with an SSFS stage based on a photonic crystal rod to generate pulses ranging from 1660 nm to 2000 nm, with pulse energies reaching up to 140 nJ^16^. A $\hbox {BiB}_3\hbox {O}_6$ crystal was used to achieve SHG from 26.4 nJ to 80 nJ at 1000 nm. Owing to the high pulse energy of the soliton, the conversion efficiency exceeded 50%.

Using commercial MgO:PPLN for SHG, Stachowiak D. *et al.* generated a SHG from an Er-doped fiber laser with SSFS to 2150 nm, followed by an SHG module utilizing a PPLN crystal with multiple quasi-phase-matching (QPM) periods to generate frequency-doubled radiation tunable from 872 to 1075 nm^25^. The setup generated a frequency-doubled beam tunable from 872 to 1075 nm, emitting sub-230 fs pulses across the tuning wavelength range. Despite high average power stability, up to 0.1% root mean square (RMS), the output power was modest, ranging from 0.68 to 1.24 mW, equivalent to pulse energies of 13.2 to 24.1 pJ. All these methods indicate that high pulse energy can be attained, however, an Er-laser with extremely high peak power is required.

In this paper, we present the development and performance results of a compact femtosecond laser source capable of generating tunable wavelengths within the first and third biological windows. This laser system is specifically designed to address the requirements for bladder cancer detection using multiphoton microscopy^7^. The system covers a spectral range of 1625 - 1700 nm, achieved through a spectrally shifted Er-doped fiber laser, and incorporates a second-harmonic generation stage based on a PPLN crystal. The developed laser emits sub-100 fs pulses, offering tunable spectral ranges of 1625 - 1700 nm with a maximum pulse energy of 10.4 nJ, and 800 - 850 nm with a maximum pulse energy of 5.1 nJ. The system achieves a conversion efficiency of up to 55% at 1680 nm, with the SHG power stability maintained within 2% RMS.

## Experimental setup

**Fig. 1:**
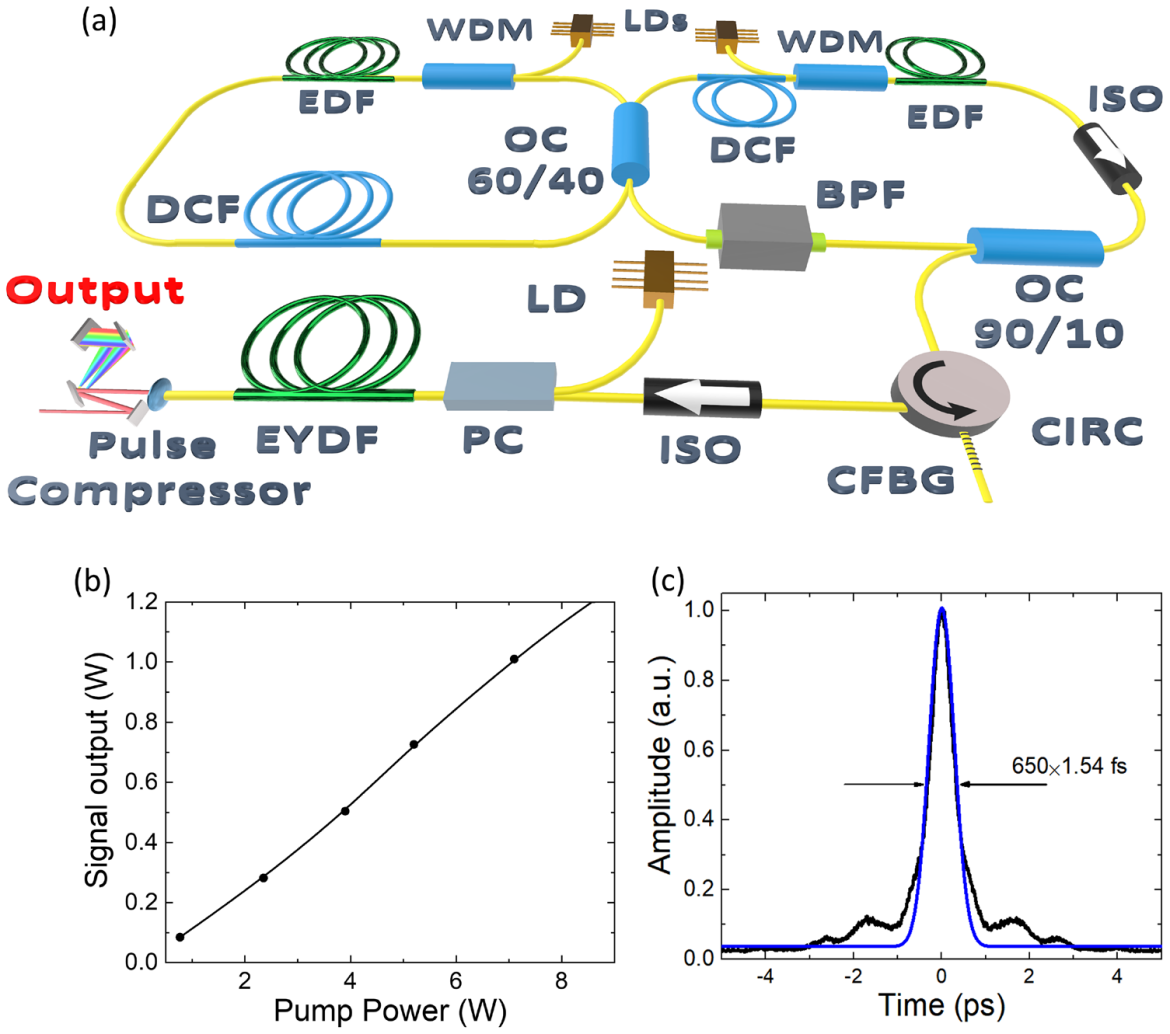
(**a**) Scheme of the Er-doped fiber laser, where LD - laser diodes, WDM - fiber wavelength division multiplexer, DCF - dispersion-compensating fiber, OC - optical coupler, CFBG - chirped fiber Bragg-grating pulse stretcher, ISO - isolator, CIRC - optical fiber circulator, PC - pump combiner, EYDF - double-clad Er/Yb co-doped fiber; (**b**) Output power of main amplifier versus pump power and (**c**) Autocorrelation trace of compressed pulse after grating pair.

The laser system, shown in Fig. 1a, consists of a custom-built, ultrafast, all-polarization-maintaining (PM), Erbium-doped (Er) fiber laser operating at a wavelength of 1550 nm. The all-PM mode-locked oscillator is characterized as a self-starting, robust system resistant to external influences. It is an 8-figure fiber master oscillator scheme based on a Nonlinear Amplified Loop Mirror serving as an artificial mode-locker^26^. The oscillator outputs an average power of $1.62\,\hbox {mW}$, corresponding to a single pulse energy of $0.15\hbox { nJ}$ at a measured pulse repetition rate of $10.9\hbox { MHz}$. The pulse is stretched from $2.3 \hbox { ps}$ to $28.6 \hbox { ps}$ in the chirped fiber Bragg grating (CFBG) stretcher, with a total dispersion of $-4.29 \hbox { ps nm}^{-1}$. Prior to entering the power amplifier, an isolator with a blocked fast axis of polarization ensures linear polarization of the pulse. The pulses are then amplified in a PM double-clad Er/Yb co-doped fiber (EYDF), with a $10\,\upmu \hbox {m}$ core diameter, pumped by high-power 980 nm laser diode (LD). The system achieves a maximum output power of 1.2 W at a pump power of 8 W. Figure 1b illustrates the dependence of the output power of the main amplifier on the pump power of the developed Er-doped high-power amplifier. The pulse exiting the power amplifier, with a duration of approximately 27 ps, is compressed using a compression system consisting of a pair of diffraction gratings (1000 lines per mm) set at a Littrow angle of $49.9^{\circ }$. The linear polarization minimizes radiation losses at each grating to less than 5%. By adjusting the distance between the gratings, we achieved a compressed pulse duration of 650 fs, which is quite close to the transform-limited duration, as gratings could compensate only second-order dispersions but not higher-order. The full width at half maximum (FWHM) of the spectra was measured as 6.1 nm, which means that the transform limit of the pulse duration (Gaussian) is around 600 fs. Further adjusting the gratings did not provide the compression of the pulse but destroyed its shape at the autocorrelator. The autocorrelation trace of the compressed pulse, measured by autocorrelator (Femtochrome FL103XL), is shown in Fig. 1c.

A Raman shift was induced within a 1-meter span of commercially available Endlessly Single Mode Large Mode Area Photonic Crystal Fiber (PCF LMA20 from NKT Photonics), boasting an effective area ($\hbox {A}_{eff}$) of $230\,\upmu \hbox {m}^2$ at 1550 nm.

**Fig. 2:**
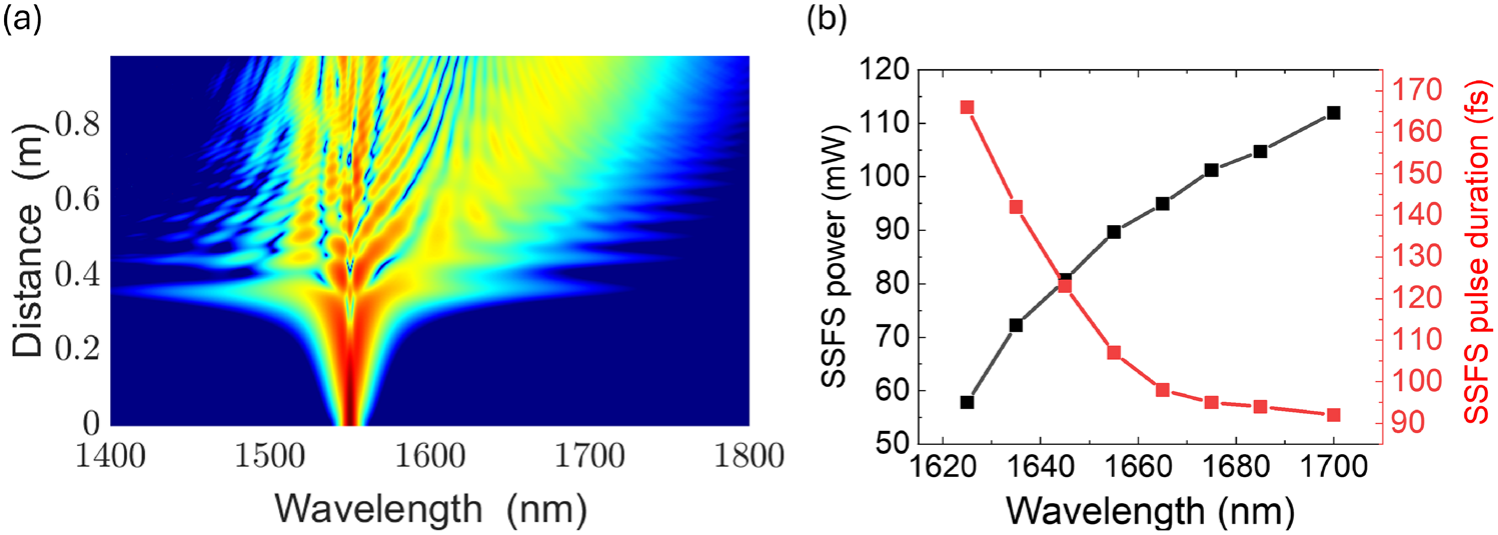
(**a**) Simulation results of soliton frequency shifting along the LMA fiber, (**b**) Dependence of SSFS power and pulse duration versus SSFS central wavelength.

The simulation results indicate that a self-soliton wavelength shift to 1700 nm can be achieved with an input power of 300 mW. Accordingly, the average output power of the developed Er-doped laser system was targeted to reach this power level after the compression stage. This corresponds to an amplifier output pulse energy of approximately 27 nJ and a peak soliton power of about 36 kW. The spectral evolution within the large-mode-area (LMA) fiber is illustrated through the simulation results presented in Fig. 2a. The modeling was performed using the initial pulse parameters measured at the output of the diffraction gratings, which include a pulse duration of 660 fs, an average power of 300 mW, a pulse repetition rate of 10.9 MHz, and a chirp value of 0. Consequently, we have generated ultrashort pulses with central wavelengths that are tunable from 1625 to 1700 nm. This spectral adjustment of the soliton central wavelength is achieved by varying the optical power injected into the PCF, specifically by regulating the pump power of the Er-doped fiber laser. An aspheric lens with a focal length of 11 mm facilitated a coupling efficiency of 60%. A recent publication has provided detailed analyses regarding the SSFS in LMA20^27^.

The resultant SSFS output power escalates concurrently with the increase in coupled power, reaching the maximum of 115 mW with a pulse duration of 92 fs at 1700 nm, corresponding to a pulse energy of 10.4 nJ. Additionally, the pulse duration of the SSFS pulse decreases as the central wavelength shifts to a longer wavelength. This dynamic relationship is effectively illustrated in Fig. 2b, showcasing the dependence of both SSFS power and pulse duration on the SSFS central wavelength.

**Fig. 3:**
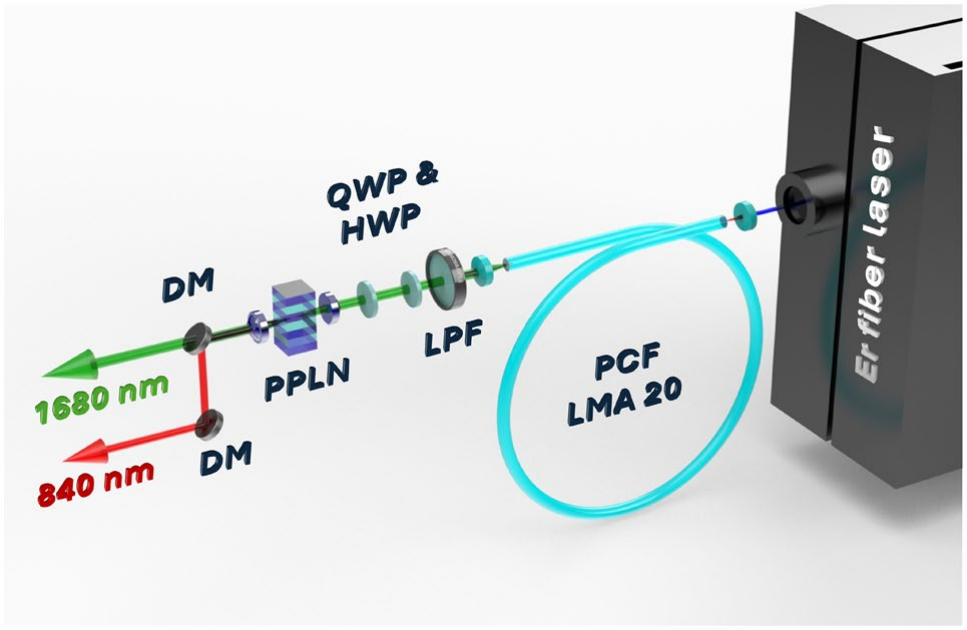
Optical layout of the fiber laser system. LPF - long pass filter 1600 nm, QWP - quarter-wave plate, HWP - half-wave plate, PPLN - periodically poled lithium niobate, DM - dichroic mirror.

The emergent beam from the SSFS stage is collimated using an additional aspheric lens with an 11 mm focal length and subsequently filtered through a long-pass filter with a cut-off wavelength near 1600 nm to suppress any residual radiation at $1.55\upmu \hbox { m}$. The polarization of the shifted soliton radiation is marginally elliptical; thus, quarter- and half-wave plates are employed to attain the polarization state required for SHG. For the SHG process, the aspheric lens with an 11 mm focal length concentrates the beam within a PPLN crystal provided by HC Photonics Corp.

According to the quasi-phase matching condition:

1 $$\begin{aligned} \begin{array}{cc} \Delta \beta _{QPM} = k_3 - k_1 - k_2 - K_{QPM}, & k_1 = k_2 = \frac{2\pi n_1}{\lambda _1}, \\ k_3 = \frac{2\pi n_3}{\lambda _3}, & K_{QPM} = \frac{2\pi }{\Lambda }. \end{array} \end{aligned}$$

The refractive indices $n_1$ and $n_3$ denote the optical indices of PPLN for the pump radiation and the SHG, respectively, while $\Lambda$ represents the QPM period. The fundamental wavelength is denoted by $\lambda _1$, and $\lambda _3$ signifies the wavelength of the frequency-doubled radiation. A PPLN crystal, characterized by a poling length of 0.3 mm and encompassing 10 QPM periods ranging from 20.6 to $23.3 \upmu \hbox {m}$, was employed to achieve high conversion efficiency across the entire wavelength tuning range. The crystal was mounted on a linear stage, oriented perpendicularly to the beam path, to facilitate precise positioning for each QPM period, thereby allowing fine-tuning for each wavelength. A temperature-controlled oven enabled tuning of the PPLN crystal temperature over a range from $20^{\circ }\hbox {C}$ to $70^{\circ }\hbox {C}$. During operation, temperature tuning between $25^{\circ }\hbox {C}$ and $35^{\circ }\hbox {C}$ was performed to achieve quasi-phase-matching for different fundamental wavelengths within the 800-850 nm tuning range. For optimal SHG efficiency at the central wavelength of 1680 nm, the PPLN crystal was maintained at $30.5 \pm 0.1^{\circ }\hbox {C}$. Subsequent to the crystal, the doubled radiation was collimated using an aspheric lens with a focal length of 11 mm. To segregate the frequency-doubled radiation from the pump signal, which spans 1625-1700 nm, two long-pass dichroic mirrors were incorporated into the setup. Moreover, a bandpass filter centered at 830 nm, with a 50 nm bandwidth, was utilized to attenuate any parasitic third and fourth harmonics produced within the PPLN crystal. The configuration of the laser system is illustrated in Fig. 3.

**Fig. 4:**
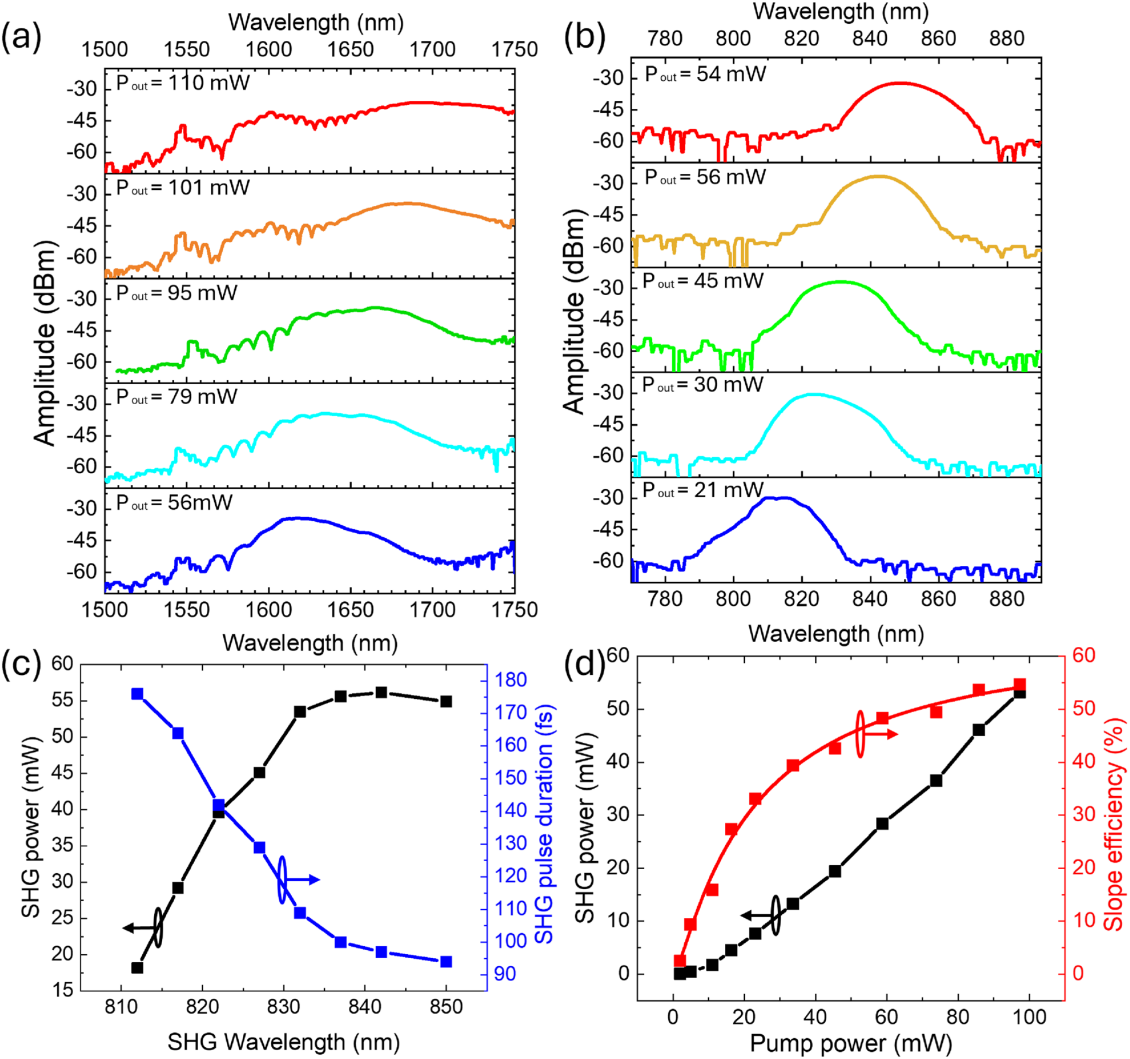
(**a**) Optical spectra of the SSFS and (**b**) corresponding SHG spectra with indicated average output powers; (**c**) dependence of maximum achieved SHG power and pulse duration versus central wavelength; (**d**) SHG output power and slope efficiency versus pump power for the case of maximum conversion efficiency at a wavelength of 840 nm.

### Results and discussion

**Fig. 5:**
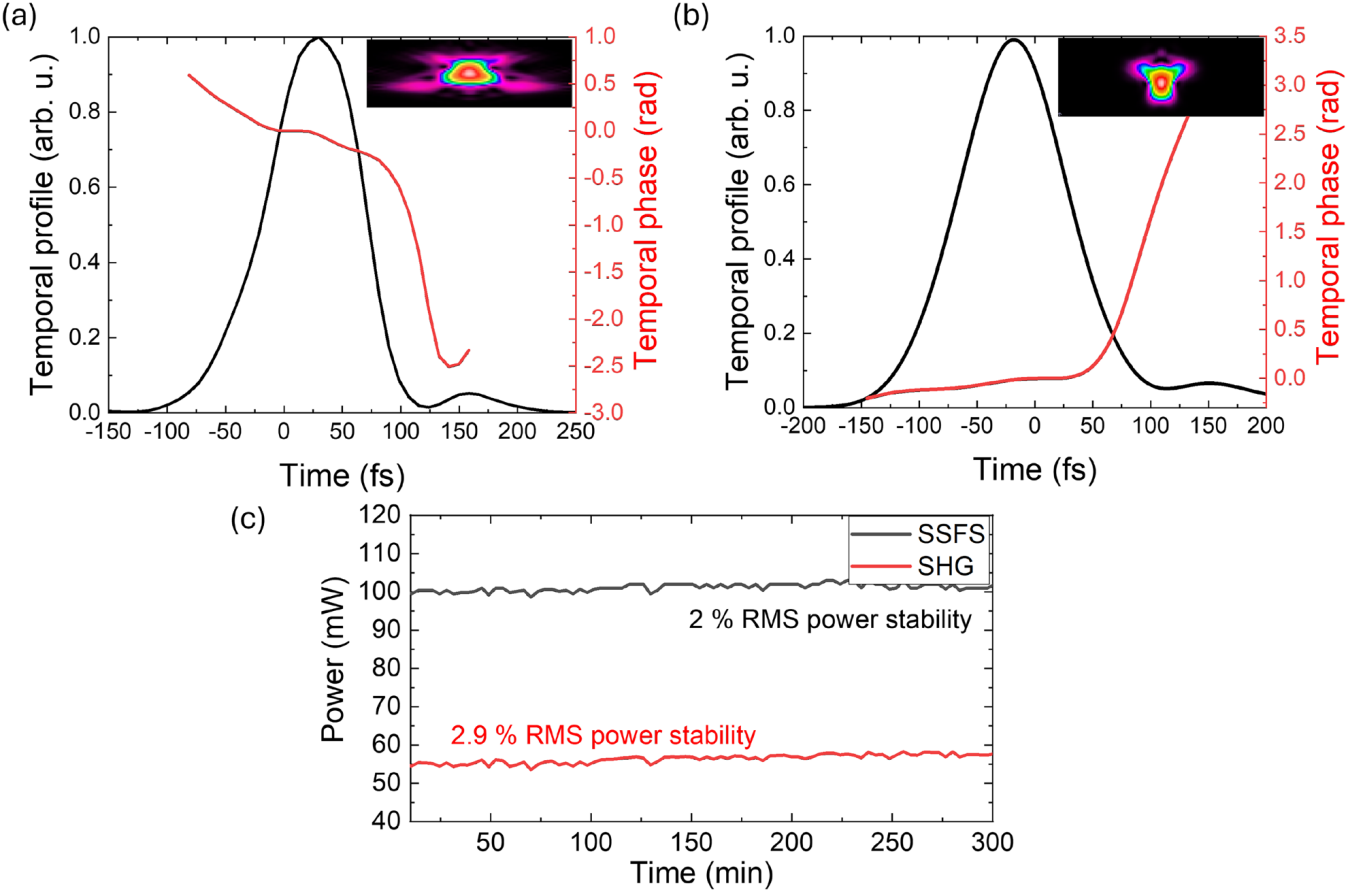
(**a**) Temporal profile of the initial SSFS pulse at 1680 nm and (**b**) temporal profile of the frequency-doubled pulse at 840 nm, with the corresponding FROG traces retrieved for each pulse; (**c**) long-term power stability measurements of the SSFS and SHG outputs.

It was found that SSFS pulses at longer wavelengths exhibit increased power. This is attributed to the increased peak power of the seeded Er-doped fiber laser coupled to the PCF. The dispersion curve of the PCF LMA20 demonstrates a consistent increase in anomalous dispersion, ranging from 25 to 50 ps/km/nm, for wavelengths extending from 1500 to 1800 nm. Consequently, at these longer wavelengths, soliton pulse durations are shorter, resulting in a broadening of the soliton spectrum.

Figure 4 a and b display the spectrum of SSFS radiation and the corresponding SHG spectrum under varying seeded laser pump powers, as recorded by an optical spectrum analyzer (Ando AQ-6315E). The SHG pulse duration was measured using an autocorrelator (APE Mini TPA, APE GmbH). A maximum second-harmonic generation output power of 56 mW, with a pulse duration of 97 fs, was achieved at a wavelength of 840 nm, as shown in Fig. 4c. To obtain this 56 mW SHG output, a 1550 nm laser operating at 300 mW was used, producing a SSFS pulse of 101 mW at 1680 nm. This resulted in an SHG conversion efficiency of over 55%, yielding an observed output power of approximately 56 mW. Figure 4d illustrates the relationship between SHG power and conversion efficiency as a function of pump power at the central wavelength of 1680 nm. At this wavelength, the conversion efficiency peaks at 55%, which is noteworthy given the relatively low pump power utilized. As indicated in Fig. 4d, the conversion efficiency does not reach full saturation at the maximum pump power, suggesting the potential for further increase with higher pump intensities. However, the available pump power was constrained by the specifications of the developed Er fiber laser, and the beam diameter was optimized to the minimum necessary to maintain uniform distribution within the crystal.

The second-harmonic generation of SSFS radiation, centered at 1680 nm, produced pulses at 840 nm. These sub-100-femtosecond pulses exhibit an energy of 5.1 nJ. Figure 5 presents the temporal profiles and phases of both the SSFS and frequency-doubled pulses. The SHG power observed when pumping at 1700 nm was slightly lower than that at 1680 nm, despite the higher peak power of SSFS at 1700 nm. This discrepancy is attributed to decreased SHG efficiency in the PPLN crystal for wavelengths exceeding 1690 nm.

A significant observation was the fission of the 1550 nm seeded pulse into a second SSFS pulse at higher pump power, occurring in the LPF transparency band beyond 1600 nm. This second pulse, at a wavelength of 1600 nm, exhibited a lower peak power compared to the 1700 nm pulse and was not efficiently converted in the PPLN crystal. It is important to note that the QPM condition varies with wavelength, making it impossible to achieve high-frequency conversion efficiency simultaneously for both solitons.

The spectral FWHM of the SHG pulses varies from 10 nm to 15 nm as the central wavelength shifts toward longer wavelengths (Fig. 4b). Correspondingly, the pulse duration also changes (Fig. 4c). The comparison between the temporal profile of the initial pulse at 1680 nm and the temporal profile of the frequency-doubled pulse at 840 nm is shown in Fig. 5 a and b. Accordingly, the widest pulse spectrum width of 15 nm has been demonstrated in the scheme based on a PPLN crystal. The high power of the generated output pulse, along with a wide wavelength tuning range, indicates the high efficiency of frequency doubling for wideband femtosecond pulses.

The long-term average power stability of the SSFS at 1680 nm and SHG at a wavelength of 840 nm was measured over 5 hours (Fig. 5 c). After the SSFS stage, the output maintained a stability of approximately 2% RMS. The SHG output exhibited slightly higher fluctuations, with a measured stability of 2.9% RMS. This gradual increase in RMS fluctuations is attributed to the introduction of free-space sections after the fiber stages, where the number of bulk optical elements increases at each stage. Environmental perturbations such as thermal drifts and mechanical vibrations in the free-space optical path contribute to the slightly reduced stability compared to the all-fiber oscillator output. The beam quality of the second-harmonic output after the PPLN crystal was measured to have $\hbox {M}^{2}$ values of 1.04 and 1.06 in the horizontal (x) and vertical (y) directions, respectively, indicating a near-Gaussian beam profile with minimal deformation. All components of the laser were operating under room temperature conditions and were located on the optical table. During the measurements, the room temperature was maintained within a range of 20-22 °C, however, temperature fluctuations could still influence the results. The laser power stability could be further improved by implementing temperature stabilization for the entire laser system.

## Conclusion

The developed tunable femtosecond laser stands out by emitting sub-100-fs pulses within a tunable spectral range of 800 nm to 850 nm, coupled with a remarkable pulse energy of 5.1 nJ in a compact design. Notably, it demonstrates a conversion efficiency of $55 \%$ at 1685 nm, which, to the best of our knowledge, represents the first instance of such high efficiency in SHG from an Er laser with SSFS in PPLN crystals. Additionally, the SHG power remains impressively stable, with fluctuations kept below $2\%$ RMS. The developed tunable femtosecond laser addresses the critical need for efficient and precise imaging tools in multiphoton microscopy, particularly for applications in bladder cancer detection. A distinctive feature of the system is its ability to generate two ultrashort pulses at different, tunable central wavelengths, enabling flexible excitation schemes for advanced biomedical imaging. The outcomes of this study not only advance biomedical imaging but also open up opportunities for the use of compact, high-efficiency lasers across a wide range of applications.

## Data Availability

The datasets used and/or analysed during the current study are available from the corresponding author on reasonable request.
